# Does grandchild care affect ageing satisfaction? Findings based on a nationally representative longitudinal study

**DOI:** 10.1371/journal.pone.0265600

**Published:** 2022-03-17

**Authors:** Eleanor Quirke, Hans-Helmut König, André Hajek

**Affiliations:** Department of Health Economics and Health Services Research, Hamburg Center for Health Economics, University Medical Center Hamburg-Eppendorf, Hamburg, Germany; University of Alberta, CANADA

## Abstract

**Objective:**

This study seeks to explore the association between grandchild care and Attitudes Towards Own Ageing, assessing whether the commencement of, or ceasing, grandchild care is associated with changes in grandparents’ perspectives on ageing.

**Methods:**

Longitudinal data were drawn from a population-based sample of community-dwelling individuals aged ≥40 years in Germany. The Attitudes Toward Own Ageing subscale of the Philadelphia Geriatric Center Moral Scale (PGCMS) was used to measure Attitudes Towards Own Ageing. To determine whether respondents provided grandchild care, respondents were asked *“I’d now like to go on to learn more about your activities and pastimes*. *Do you supervise other people’s children privately*, *e*.*g*. *your grandchildren*, *or the children of siblings*, *neighbors*, *friends or acquaintances*?*”* Symmetric and asymmetric linear fixed effects regressions were used to assess within-person changes longitudinally.

**Results:**

No statistically significant association between providing care for grandchildren and Attitudes Towards Own Ageing was found. Significant associations were found between Attitudes Towards Own Ageing and employment status. Namely, retirement was associated with more positive Attitudes Towards Own Ageing (β = 0.57, p < .001), as was not being employed (β = 0.57, p < .001). A significant association between self-rated health and Attitudes Towards Own Ageing was also found (β = -0.06, p < .001), with poorer self-rated health associated with more negative Attitudes Towards Own Ageing.

**Conclusion:**

Our findings suggest that undertaking grandchild care does not shape Attitudes Towards Own Ageing. As our findings did not align with existing evidence on the associations between grandchild caregiving and measures of subjective ageing, further research is required.

## 1. Introduction

Demographic ageing poses a number of questions for policymakers across the world, including how best to support individuals to stay independent and active as they age [1]. The World Health Organization adopted the term “active ageing” to express the process of maintaining a high quality of life, including good health, social participation and security, as individuals age, stating that interventions that create supportive environments and foster healthy choice are important at all stages of life [1].

Over the past 40 years, a large amount of research has been undertaken on the ageing process, including research into experiences of the ageing process and perceptions of age. A number of studies have demonstrated that age is more than a number (or one’s chronological age). Rather, age is a construct that is rooted in personal experiences, and has social and personal meanings [2]. Research on the subjective aspects of age and the ageing process has encompassed a range of similar but distinct concepts, including subjective age, age identity, self-perceptions of ageing and attitudes towards ageing. An outline of these concepts, based on reviews of the constructs of subjective ageing undertaken by Diehl et al., 2015, Kotter-Grühn et al., and Diehl et al., 2014 [2–4], can be found in S1 Appendix. We note that the terms self-perceptions of ageing, Attitudes Towards own Ageing and satisfaction with ageing are used interchangeably. In this article, we explore perspectives on the experience of ageing measured using the Attitudes Towards own Ageing subscale of Philadelphia Geriatric Center Moral Scale.

The importance of subjective experiences of ageing is demonstrated by its association with a range of health outcomes, including mortality. For example, Uotinen et al. found that perceived age predicted mortality [5], while Markides & Pappas found, perceived age to be a better predictor of survival than life expectancy [6]. Similarly, individuals with more positive Attitudes Towards own Ageing have been demonstrated to maintain better functional health [7], and positive Attitudes Towards own Ageing have also been found to have a preventive effect on basic and instrumental activities of daily living, falls and hospitalizations [8]. Feeling younger than one’s chronological age has been shown to have significant positive effects on physical health, cognitive functioning and longevity [5, 9–12]. Furthermore, among older people, youthful age identities are related to higher levels of morale and life satisfaction [13]. On the other hand, dissatisfaction with one’s age and feeling older than one is chronologically has been linked to increased mortality [5], whilst negative age stereotypes (namely, negative beliefs among older people about older people) have been associated with lower cognitive functioning and poorer functional health [14–16].

In view of the direct implications that subjective perceptions of age have for wellbeing and survival, researchers have called for further research into the subjective aspects of ageing. Indeed, as Kotter-Grühn et al. noted in their 2016 study, “asking individuals how old they feel may seem trivial. Yet their responses yielded powerful insights into both physical and psychological ageing” [4]. Specifically, examining if people traditionally considered ‘old’, do indeed feel old, and when they begin to do so, is of crucial importance, as such insights can deepen understanding of how best to support individuals as they age.

Research to date has identified a range of correlates of these subjective experiences of ageing. For example, physical and psychological health status have been found to be consistent correlates of self-perceptions of ageing [4]. Optimism, higher self-efficacy, higher conscientiousness and higher levels of personal growth and generativity have been demonstrated to contribute to a lower perceived age [17–19]. Social networks have also been shown to reduce the importance of age among older individuals; individuals identify themselves in relation to the roles they fulfill, or the tasks they undertake as part of these roles, rather than by their age [20].

In line with the notion that experiences of age are rooted in personal experience, social and cultural systems and norms, social roles and role transitions (i.e. parenthood, marriage retirement, widowhood) have been conceptualized as signposts of ageing, shaping the “rhythm of the life course”, as well as the daily life, thereby shaping perceptions of ageing (p453, [21]). This article seeks to build on the literature on the role of role transitions on subjective ageing, by exploring the role of grandparenthood and grandchild caregiving in Attitudes Towards own Ageing. Grandparenthood has been characterized as a powerful reminder of a person’s ageing [22], and can arguably be considered a significant milestone in life, like retirement. Research to date has found that being a grandmother is an important factor for age identity, as is the number of grandchildren one has [23]. Those who enjoy being a grandparent have been found to feel younger, perceive old age later and hope to live longer [23]. The timing of entry into grandparenthood has also been found to be a pertinent correlate of age identity. Those who transition to grandparenthood earlier than their peers feel older than those who’d transitioned to grandparenthood ‘on time’ [23].

Grandparenthood is unique, in that it provides an opportunity for older individuals to be actively involved in the care of, and be in contact with, with younger children [24]. It is plausible therefore, that in addition to becoming a grandparent, the act of grandchild caregiving, a key task associated with the role, may shape ageing experiences among grandparents. The provision of grandchild care and subjective ageing have been examined in two studies to date. The first study was a cross-sectional study conducted in 2016. Drawing on data from a population-based sample (namely from the Health and Retirement Study), the study found that grandparents feel older than their grandchildless counterparts, but that this is reversed in later life when they look after their grandchildren [24]. A 2017 longitudinal study sought to replicate these analyses using waves 2–4 of the German Aging Survey (DEAS), and also extended the analysis to also include the provision of care to other children (i.e. the provision of childcare to other children, other than the grandparent’s grandchild). Specifically, the study compared the subjective ages of grandparents who cared for their grandchildren, and those who did not, and explored whether the provision of care to other children had the same effect as the care of a grandparent’s own grandchild. This study found that looking after grandchildren is associated with lower subjective age in life. Furthermore, the study found that the provision of care for other children was associated with a youthful subjective age, no less with similar effect sizes generated by engaging in the provision of grandchild care [22].

This present study seeks to build on the findings of the above-described studies, and in particular, the study conducted by Bordone [22]. Our study will draw on the same base data, namely the DEAS study. However, this study considers a different aspect of subjective ageing, namely Attitudes Towards own Ageing. Moreover, this study will use a different methodology, in order to exploit the panel data collected as part of the DEAS. Specifically, the methodology of this study draws on a recent study that assesses the longitudinal association between grandchild care and health and wellbeing, undertaken by Danielsbacka et al. (2019) [25]. In the Danielsbacka et al. study, two separate analyses were conducted; the first analysis assessed the association between grandchild care and health and wellbeing across participants over time, and the second analysis assessed the association between the variables of interest within individuals over time. Danielsbacka et al. (2019) found that associations between the variables did not hold, once within-person variation was analyzed, suggesting only a limited association between grandchild care and grandparental well-being, that “may be specific to physical rather than cognitive factors” [25]. They conclude that such an analysis provided a more nuanced picture of the associations between the variables of interest, to determine whether there is a causal nature to the associations [25]. In view of these findings, this study will undertake fixed effects (FE) regression analyses to observe changes within individuals over time, assessing whether the commencement or ceasing of grandchild care was associated with subsequent changes in grandparents’ perception of ageing.

In sum, this study will complement and build upon existing literature that seeks to quantify the impact of life milestones on experiences of ageing, by exploring the role of the commencement and ceasing of grandchild caregiving on Attitudes Towards own Ageing, thereby deepening understanding of the conditions under which individuals feel younger.

## 2. Materials and methods

### 2.1 Sample

This study used data drawn from the German Ageing Survey (DEAS), funded by the Federal Ministry for Family Affairs, Senior Citizens, Women and Youth. The survey is population-based and considers the experience of ageing among individuals aged 40 years and over living in private households. Topics included in the survey include employment and occupation, health, as well as social aspects, for example, feelings of loneliness.

The DEAS has a cohort-sequential design comprising of cross-sectional samples and panel samples. In addition to surveying the initial baseline cohort, new samples are added at each new wave of the survey. To date, the DEAS has consisted of six waves.

The survey itself consists of a structured personal interview conducted in the respondents’ home (capturing, for example, socio-demographic information) as well as a self-administered questionnaire completed by the respondent themselves (capturing health data, as well as more sensitive data related to sexuality, for example).

All respondents are required to give written consent prior to participation in the survey. Furthermore, the survey is overseen by a permanent advisory board, who review the sampling method as well the various measurements used prior to the commencement of each new way of the survey. This board determined prior to the commencement of each wave that ethics committee approval was not required, as the criteria for such was not met (e.g. examination of patients, risks for respondents).

In this study, data from the fourth (2011), fifth (2014) and sixth (2017) waves of the survey was analyzed. The response rate for each wave was 56%, 33% and 63% respectively.

### 2.2 Dependent variables

Attitudes Towards own Ageing were measured using the Attitudes Toward Own Ageing subscale of the Philadelphia Geriatric Center Moral Scale (PGCMS). This comprises four statements, with which respondents are asked to agree (scored 0) or disagree (scored 1). The statements are: “Things keep getting worse as I get older”; “I have as much pep as I did last year”; “As you get older, you are less useful”; “As I get older, things are better than I thought they would be”; “I am as happy now as I was when I was younger”. Three items were recoded. Items were averaged to create the final score. The total score ranges from 1 to 4, with higher values reflecting more positive attitudes towards own ageing.

### 2.3 Independent variables

To determine the commencement or ceasing of caregiving, respondents were asked *“I’d now like to go on to learn more about your activities and pastimes*. *Do you supervise other people’s children privately*, *e*.*g*. *your grandchildren*, *or the children of siblings*, *neighbors*, *friends or acquaintances*?*”*. Those who responded that they supervised their grandchildren, or their grandchildren and other children, were coded as 1 (otherwise they were coded as 0). Those whose code changed from 0 to 1 during the course of the three waves were categorized as having commenced grandchild care. In contrast, those whose code changed from 1 to 0 during the course of the three waves were categorized as having ceased grandchild care.

### 2.4 Potential confounders

A number of variables were controlled for in the analyses. These included age, marital status (married and living together with spouse and other (divorced, widowed, single, living separately to spouse)), household net equivalent income (measured in Euros), employment status (employed; retired; other), total number of physical illnesses (cardiac and circulatory disorders; bad circulation, joint, bone, spinal or back problems; respiratory problems, asthma, shortness of breath; stomach and intestinal problems; cancer; diabetes; gall bladder, liver or kidney problems; bladder problems; eye problems, vision impairment; ear problems, hearing problems), self-rated health (1 = very good to 5 = very bad) and physical functioning (subscale of the SF-36 with scores ranging from 0 to 100; higher values refer to a higher degree of physical functioning).

### 2.5 Statistical analysis

Descriptive statistics for participants (pooled) were first calculated. Following this, linear FE regressions were used to estimate the effect of time-varying explanatory variables (in this case, the commencement of or ceasing of grandchild care) on the dependent variables (perception of ageing), allowing for changes within individuals over time to be observed [26].

Three separate models were built. The first model analyzed whether an association between the commencement of grandchild care and Attitudes Towards own Ageing exists, irrespective of the direct of change, i.e. irrespective of whether the grandparent had begun caring for their grandchildren or had since ceased. The second model computed the association between the having begun to provide care to a grandchild (i.e. the model included the change from not caring for grandchildren to then undertaking care of the grandchild) and Attitudes Towards own Ageing. The third model computed the association between ceasing caring for grandchildren (i.e. the model included the change from caring for grandchildren to ceasing care) and Attitudes Towards own Ageing. Such an approach to panel data analysis is termed asymmetric FE regressions, and was first introduced by Allison [27].

## 3. Results

Sample characteristics for the analytical sample (N = 3118) can be found in Table 1. In summary, 46.2% of the sample was female. The average age of the sample was 74.8 years, and 71.5% of the same was married and living together with their spouse. The average number of physical illnesses was 3.2, and the self-rated health score was 2.5 (1 = very good to 5 = very good).

**Table 1:** Characteristics of the analytical sample (n = 3118 observations).

	N/mean	%/(SD)
Gender: female	1,440	46.2%
Age in years	74.8	5.7
Marital status: married and living together with spouse	2,229	71.5%
Employment status: retired	3,042	97.6%
Employment status: employed	67	2.2%
Employment status: other; not employed.	9	0.3%
Educational status: high education	1,374	44.1%
Educational status: medium education	1,550	49.7%
Educational status: low education	194	6.22%
Monthly net equivalent income in Euro	1870.0	1227.6
Health related quality of life (physical functioning subscale of the SF-36, ranging from 0 (worst) to 100 (best))	78.3	23.5
Number of physical illness (0–11)	3.2	2.0
Self-rated health (1 = very good to 5 = very bad)	2.5	0.8

Of the analytical sample, 330 individuals began providing grandchild care (for example, had not cared for grandchildren in 2014, but was caring for grandchildren in 2017). In addition, 381 ceased providing grandchild care (for example, had cared for grandchildren in 2011, but was not caring for grandchildren in 2014).

### 3.1 Regression analysis

Results from the FE regression analyses can be found in Table 2. In all three models, no statistically significant association between providing care for grandchildren and Attitudes Towards own Ageing was found. That is, no association was found between Attitudes Towards own Ageing and commencing, as well as ceasing, to provide grandchild care.

**Table 2:** Determinants of self-perceptions of aging. Results of fixed effects regressions (waves 4, 5 and wave 6).

	(1)	(2)	(3)
VARIABLES	Self-perceptions of aging	Self-perceptions of aging	Self-perceptions of aging
Age	-0.02***	-0.02***	-0.02***
	(0.00)	(0.00)	(0.00)
Marital status:—Other (single; divorced; widowed; married, and living separated from spouse (Ref.: married, and living together with spouse)	-0.07	-0.05	-0.08
	(0.06)	(0.06)	(0.06)
Physical functioning (Physical functioning subscale of the SF-36; ranging from 0 (worst) to 100 (best))	0.00**	0.00**	0.00**
	(0.00)	(0.00)	(0.00)
Employment status:—Retired (Ref.: Employed)	0.57***	0.57***	0.52***
	(0.09)	(0.09)	(0.09)
- Other: not employed	0.58***	0.61***	0.53***
	(0.12)	(0.12)	(0.12)
Total number of physical illnesses (ranging from 0 to 11)	-0.01	-0.01	-0.01
	(0.01)	(0.01)	(0.01)
Household net equivalent income (in Euro)	-0.00	-0.00	-0.00
	(0.00)	(0.00)	(0.00)
Self-rated health (from 1 = very good to 5 = very bad)	-0.06***	-0.07***	-0.06***
	(0.02)	(0.02)	(0.02)
Change in grandchild care (direction not specified)	-0.00		
	(0.03)		
Began undertaking grandchild care		0.02	
		(0.04)	
Ceased undertaking care			-0.04
			(0.04)
Constant	4.02***	4.18***	4.22***
	(0.32)	(0.34)	(0.34)
Observations	3,118	2,747	2,885
Number of individuals	1,625	1,406	1,490
R^2^	0.0651	0.0753	0.0711

Beta-coefficients were reported; Cluster-robust standard errors in parentheses

*** p<0.001

** p<0.01, * p<0.05, + p<0.10.

In all three models, significant associations between employment status and Attitudes Towards own Ageing were identified. Namely, retirement was associated with more positive Attitudes Towards own Ageing scores (β = 0.57, p < .001), as was not being employed (β = 0.57, p < .001). Furthermore, a significant association between self-rated health and Attitudes Towards own Ageing was found (β = -0.06, p < .001), with poorer self-rated health associated with more negative Attitudes Towards own Ageing.

## 4. Discussion

This study sought to identify how grandparent caregiving shaped Attitudes Towards own Ageing. In addition, this study sought to identify whether commencing or ceasing grandparent care influenced Attitudes Towards own Ageing among grandparents. No statistically significant associations between these variables of interest were found in a large community-based sample of adults ≥ 40 years in Germany. Therefore, our study’s findings seem to suggest that grandchild caregiving does not shape experiences of ageing; namely, how individuals experience the gains and losses associated with ageing.

To the best of our knowledge, this study is the first to assess the association between grandchild caregiving and Attitudes Towards own Ageing. Two previous studies, namely one cross-sectional study by Bordone and Arpino in 2016, and one longitudinal study by Bordone in 2017, assessed the association between *subjective age* and grandchild caregiving. Bordone and Arpino’s cross-sectional study found that taking care of grandchildren is associated with younger subjective age both for grandfathers and grandmothers [24]. Bordone’s longitudinal study found that grandparents looking after their grandchildren feel at least 2 years younger than their counterparts not engaging in grandparental childcare [22].

Why is it that grandparent caregiving influences how old a person feels, but not how they experience and perceive the ageing process? It is difficult to find an intuitive explanation. Over and above being a grandparent, the act of caregiving has been demonstrated to influence a number of social- and health-related aspects of life. Specifically, the provision of grandchild care has been linked to improved cognitive function [28], self-rated health [29] and physical health [30], as well as a greater social network size [31, 32], and reduced feelings of loneliness [31]. Furthermore, the provision of grandchild care, over and above the role of being a grandparent, has been characterized as providing an opportunity to establish and reinforce social links with others [33]. Drawing on these links, and in view of demonstrated associations between social integration, social loneliness and self-perceptions of ageing, including Attitudes Towards on Ageing [34, 35], we thought it possible that the act of grandchild caregiving could positively influence attitudes towards ageing.

These differential findings seem to underline the prior distinctions made in the literature between the concepts of subjective age and Attitudes Towards own Ageing [2, 4]. That is, the present study seems to confirm prior assertions that subjective age and Attitudes Towards own Ageing are distinct concepts, measuring different aspects of the ageing experience. Attitudes Towards own Ageing were measured in this study by asking respondents to assess their current experiences, informed by and alongside their experiences of being younger. In one item of this measure, these comparisons are made between reasonably close periods of time (i.e. I have as much pep as I did *last year*). Therefore, Attitudes Towards own Ageing may represent a more incremental assessment of the ageing process. Indeed, Attitudes Towards own Ageing have been described as an indicator for the ability to adapt to age-related losses, namely by providing insight into small adjustments and adaptations the individual makes during the ageing process. As Kotter-Grühn et al. note, to maintain positive self-perceptions of ageing, older adults may “downgrade the importance of negative age related changes in their self-concept or interpret the ways that they are dealing with these changes as positive psychological growth” [9]. On the other hand, determination of how old one feels (i.e. subjective age) requires the implicit assessment of whether one’s experience of an age matches one’s expectations of how this age would feel (i.e. it is a comparison of personal experiences and normative conceptions of ageing). Elsewhere, subjective age and self-perceptions of ageing have been differentiated in that self-perceptions of ageing measures the “affective and cognitive components of how an individual perceives the aging process, while felt age captures *a global estimation* of the feeling of one’s age”(p19, [36], emphasis added).

Applying a similar perspective to our study’s results, a possible explanation for the differential results identified by our study could be that, whilst commencing or ceasing grandchild care may on the surface appear a significant milestone or transition in the ageing process, to the individual it may represent a natural or expected change. The commencement or ceasing of grandchild care, given one has already become a grandparent, could be perceived as a matter of course, thereby having limited impact on how the ageing process is perceived. However, when considering one’s subjective age, a broader assessment of the ageing process, life changes, such as the commencement or ceasing of caregiving, may be more symbolic for the individual, or may be more imbued with personal meaning. Therefore, commencement or ceasing of grandchild caregiving may hold more influence on how old a person feels, despite perhaps having limited impact on the more short-term perceptions of the ageing experience.

This seemingly aligns with the logic underpinning Levy’s theory of the embodiment of ageing. In her theory, Levy [37] describes how the experience of arbitrary demarcations or societal cues for old age (i.e. enrolment in social security, senior admission to movie theatres) may lead older people to identify as an “old person”. Through this, Levy’s theory proposes that the individual applies negative stereotypes associated with old age to themselves, which, through psychological, physiological and behavioral pathways, can negatively impact upon the wellbeing of the individual [37]. Applying this to our study, the experience of becoming, and acting in the role of, a grandparent may cause stereotypes, or indeed positive perceptions, of grandparenthood to become self-relevant to the individual and shape how old they feel. The act of caregiving as a grandparent, however, may not have much impact on the experience of ageing, as it is a task stereotypically associated with grandparenthood. While no significant findings were identified for our association of interest, our study did identify significant associations between Attitudes Towards own Ageing and self-rated health. These significant negative associations are in line with previous studies. Namely, a number of studies have also found clear associations between physical health and subjective wellbeing, and other subjective ageing constructs [35, 38–40], with poorer self-health associated with more negative perceptions of the ageing experience.

Our study also found significant associations between not being employed and Attitudes Towards own Ageing, as well as between retirement and Attitudes Towards own Ageing. These findings are a little more difficult to relate to existing literature. Not being employed can be a reasonably heterogenous group. In the DEAS survey, not being employed includes those out of work or homemakers. Furthermore, the findings pertaining to retirement are also difficult to relate to existing literature, given mixed findings on the role of retirement and subjective experiences of ageing. For example, some studies have found that retired persons feel older than those who are still working [41–43], whilst other studies have found that retirement does not significantly influence perceived age or age identity [21, 44].

Indeed, considering these findings related to retirement, alongside the findings related to our variable of interest, we are presented with an overall murky picture on the association between social roles and life transitions in later, and Attitudes Towards own Ageing. Whilst grandparenting and retirement are distinct experiences, both mark key milestones in life, and both experiences can be associated with both gains and losses. Retirement can be perceived as gaining more free time, but also the loss of a role critical for identity. Grandparenting can be perceived as the gain of a new role and a new way to contribute to the family, but it can also be perceived as a burden and sometimes stressful task. It is clear to us that further research is required to clarify both how these two life transitions shape subjective ageing experiences longitudinally and identify the underlying mechanisms for their differing influence.

### 4.1 Strengths and limitations

The study used a large nationally representative sample, which has been found to suffer from minimal selection bias. A previously validated tool was used to measure Attitudes Towards own Ageing. FE regressions, controlling for unobserved (time-constant heterogeneity) were used to identify longitudinal associations between the variables of interest. Only a small sample selection bias has been reported for the DEAS study [45].

A limitation of our study is that grandparent care was assessed using a yes/no response regarding the private care of children that were not the respondent’s own. Our study did not differentiate between care provided to grandchildren, and the care provided to the children of neighbors or other acquaintances. Furthermore, research that considers the nature of grandchild caregiving, including its frequency and intensity, would provide additional insights into whether grandchild caregiving shapes experiences of ageing. The frequency and intensity of grandchild care has been demonstrated to mediate the impact of grandchild care and health outcomes [46], and it is feasible that these characteristics of caregiving would shape ageing experiences as well. Different generational beliefs and attitudes were not quantified in our study. Additionally, the starting age of grandparent care and the duration of care provision was not measured. Furthermore, enjoyment of grandchild caregiving, as well as the voluntary nature of grandchild caregiving, may also provide additional insights, given the role these characteristics have played in other examinations of the impact of grandparent caregiving [23, 33]. Finally, it should be acknowledged that a small attrition bias exists in the DEAS study [45], which could have slightly biased the estimates.

## 5. Conclusions

This longitudinal study, using data from a representative, population-based survey, did not identify any statistically significant associations between caring for a grandchild, as well as commencing or ceasing grandchild care, and Attitudes Towards own Ageing. Further research on the act of grandchild caregiving and its relationship with perceptions of ageing would provide further insight into the conditions that shape successful ageing.

## Supporting information

S1 Appendix Definitions of ageing concepts. (DOCX)
