# Supplementary material for: Does grandchild care affect ageing satisfaction? Findings based on a nationally representative longitudinal study
Source: PLoS One. 2022 Mar 17;17(3):e0265600. doi: 10.1371/journal.pone.0265600 (PMC8929628; doi:10.1371/journal.pone.0265600)
Supplement: S1 Appendix — (DOCX) [file pone.0265600.s001.docx]

**S1 Appendix. Definitions of ageing concepts.**

| Concept | Definition |
| --- | --- |
| Chronological age/calendar age | Age measured in years. |
| Subjective age/subjective ageing | How old a person feels or how old they see themselves [1–3]. |
| Age identity | Age identity has been distinguished from subjective age/subjective ageing in view of its roots in sociology and social identity theory [3]. It has been argued that age identity reflects an individual’s perception of age based on their social experiences, conceptions of the life course and the social roles individuals occupy throughout the life course and/or identification with a particular age group, as opposed to their chronological age [3]. Similarly, age identity has also been described as the age group or cohort to which an individual feels they belong [1]. |
| Self-perceptions with ageing | Self-perceptions of ageing is a multi-dimensional construct, that considers how people experience their ageing [1–3]. Self-perceptions of ageing are rooted in an individual’s personal experiences of age [2,3]. This construct takes into account an individual’s satisfaction with their age and the ageing process, as well as how their experience of the ageing process aligns with their expectations [1]. |
| Attitudes towards ageing and age stereotypes | Attitudes towards ageing refer to both societal and individual attitudes toward older adults and the process of ageing [2,3]. They comprise affective, cognitive and evaluative components of behavior [2,3]. Attitudes towards ageing are closely linked to age stereotypes [4], namely attitudes and reactions towards old people and the ageing process that are primarily negative [2,3]. Both age stereotypes and attitudes towards ageing are based on social, cultural and historical understandings of, and beliefs related to, ageing [2,3]. |
| Awareness of age-related change | The experiences that prompt recognition or awareness in an individual that their behavior, level of performance, or ways of experiencing life have changed as a consequence of having become older. It is a conscious perception of a change attributed to ageing [2,3]. |
